# Cryptococcal Meningitis in Kidney Transplant Recipients: A Two-Decade Cohort Study in France

**DOI:** 10.3390/pathogens11060699

**Published:** 2022-06-17

**Authors:** Laurène Tardieu, Gillian Divard, Olivier Lortholary, Anne Scemla, Éric Rondeau, Isabelle Accoceberry, Rémi Agbonon, Alexandre Alanio, Adela Angoulvant, Laetitia Albano, Philippe Attias, Anne Pauline Bellanger, Dominique Bertrand, Julie Bonhomme, Françoise Botterel, Nicolas Bouvier, Matthias Buchler, Taieb Chouaki, Thomas Crépin, Marie-Fleur Durieux, Guillaume Desoubeaux, Gary Doppelt, Loïc Favennec, Arnaud Fekkar, Ophélie Fourdinier, Marie Frimat, Jean-Pierre Gangneux, Claire Garandeau, Lilia Hasseine, Christophe Hennequin, Xavier Iriart, Nassim Kamar, Hannah Kaminski, Raphael Kormann, Laurence Lachaud, Christophe Legendre, Moglie Le Quintrec Donnette, Jordan Leroy, Charlène Levi, Marie Machouart, David Marx, Jean Menotti, Valérie Moal, Florent Morio, Natacha Mrozek, Muriel Nicolas, Philippe Poirier, Marie-Noelle Peraldi, Benjamin Poussot, Stéphane Ranque, Jean-Philippe Rerolle, Boualem Sendid, Renaud Snanoudj, Jérôme Tourret, Marc Vasse, Cécile Vigneau, Odile Villard, Laurent Mesnard, Fanny Lanternier, Cédric Rafat

**Affiliations:** 1 Service de Soins Intensifs Néphrologiques et Rein Aigu, French Intensive Renal Network, Hôpital Tenon, 75020 Paris, France; eric.rondeau@aphp.fr (É.R.); laurent.mesnard@aphp.fr (L.M.); cedric.rafat@aphp.fr (C.R.); 2 Paris Translational Research Centre for Organ Transplantation, PARCC, INSERM U970, Université de Paris, 75015 Paris, France; gillian.divard@gmail.com; 3 Department of Nephrology and Kidney Transplantation, Necker Hospital, Assistance Publique-Hôpitaux de Paris, Université Paris Descartes Sorbonne Paris Cité, 75015 Paris, France; anne.scemla@aphp.fr; 4 Centre d’Infectiologie Necker-Pasteur, Université de Paris Cité, Hôpital Necker-Enfant Malades, AP-HP, 75015 Paris, France; olivier.lortholary@aphp.fr (O.L.); fanny.lanternier@aphp.fr (F.L.); 5 Molecular Mycology Unit, National Reference Center for Invasive Mycoses and Antifungals, Institut Pasteur, Université Paris Cité, CNRS UMR20000, 75015 Paris, France; 6 Laboratory of Parasitology-Mycology, Pellegrin University Hospital, 33000 Bordeaux, France; isabelle.accoceberry@chu-bordeaux.fr; 7 Department of Radiology, Tenon Hospital, 75020 Paris, France; remi.agbonon@aphp.fr; 8 Laboratory of Parasitology-Mycology, Saint-Louis Hospital, AP-HP, 75010 Paris, France; alexandre.alanio@aphp.fr; 9 Service de Maladies infectieuses et Tropicale, APHP-Hôpital Bicêtre, 94270 Le Kremlin-Bicêtre, France; adela.angoulvant@aphp.fr; 10 Department of Nephrology and Transplantation, 06000 CHU Nice, France; albano.l@chu-nice.fr; 11 Nephrology and Renal Transplantation Department, APHP, Groupe Hospitalier Henri-Mondor, 94010 Créteil, France; attias.philippe@gmail.com; 12 Parasitology Mycology Department, University Hospital, 25000 Besancon, France; apbellanger@chu-besancon.fr; 13 Department of Nephrology and Transplantation, University of Rouen, 76000 Rouen, France; dominique.bertrand@chu-rouen.fr; 14 Laboratory of Parasitology-Mycology, CHU Caen, 14033 Caen, France; bonhomme-j@chu-caen.fr; 15 Laboratory of Mycology, AP-HP (Assistance Publique-Hôpitaux de Paris), Hôpitaux Universitaires Henri Mondor, 94010 Créteil, France; francoise.botterel@aphp.fr; 16 Department of Nephrology and Transplantation, University of Caen, 14033 Caen, France; bouvier-n@chu-caen.fr; 17 Service de Néphrologie-Hypertension, Dialyses, Transplantation Rénale, Hôpital Bretonneau et Hôpital Clocheville, 37000 Tours, France; mathias.buchler@univ-tours.fr; 18 Service de Parasitologie et Mycologie Médicales, CHU d’Amiens, 80000 Amiens, France; chouaki.taieb@chu-amiens.fr; 19 Department of Nephrology, Dialysis, and Renal Transplantation, CHU Besançon, 25000 Besançon, France; tcrepin@chu-besancon.fr; 20 Laboratoire de Parasitologie—Mycologie, CHU de Limoges, 87000 Limoges, France; marie-fleur.durieux@chu-limoges.fr; 21 Department of Parasitology-Mycology Service, Tropical Medicine Program, University Hospital of Tours, 37000 Tours, France; guillaume.desoubeaux@univ-tours.fr; 22 Department of Radiology, André Rosemon, Hôpital de Cayenne, Université des Antilles et de la Guyane Française, 97139 Pointe à Pitre, France; gary.doppelt@yahoo.fr; 23 Department of Parasitology/Mycology, Rouen University Hospital, 76000 Rouen, France; loic.favennec@chu-rouen.fr; 24 Service de Parasitologie Mycologie, Assistance Publique—Hôpitaux de Paris (AP-HP), Groupe Hospitalier Pitié-Salpêtrière, 75013 Paris, France; arnaud.fekkar@aphp.fr; 25 Department of Nephrology, Dialysis and Transplantation, CHU Amiens, 80000 Amiens, France; fourdinier.ophelie@chu-amiens.fr; 26 Nephrology Department, University Lille, CHU Lille, 59000 Lille, France; marie.frimat@chru-lille.fr; 27 Centre Hospitalier Universitaire de Rennes, Laboratoire de Parasitologie-Mycologie, Rennes, France INSERM U1085, IRSET (Institut de Recherche en Santé Environnement Travail), Université Rennes 1, 35000 Rennes, France; jean-pierre.gangneux@chu-rennes.fr; 28 Department of Nephrology and Immunology, Institut de Transplantation Urologie Néphrologie (ITUN), 44000 Nantes, France; claire.garandeau@chu-nantes.fr; 29 Centre Méditerranéen de Médecine Moléculaire, Université de Nice-Sophia Antipolis, 06000 Nice, France; hasseine.l@chu-nice.fr; 30 Centre de Recherche Saint-Antoine, CRSA, Inserm, AP-HP, Hôpital Saint-Antoine, Service de Parasitologie-Mycologie, Sorbonne Université, 75012 Paris, France; christophe.hennequin-sat@aphp.fr; 31 Department of Parasitology-Mycology, Toulouse University Hospital Toulouse, 31400 Toulouse, France; iriart.x@chu-toulouse.fr; 32 Department of Nephrology and Organs Transplantation, Toulouse Rangueil University Hospital, INSERM UMR 1291, Toulouse Institute for Infectious and Inflammatory Disease (Infinity), Paul Sabatier University, 31400 Toulouse, France; kamar.n@chu-toulouse.fr; 33 Department of Nephrology, Transplantation, Dialysis and Apheresis, Pellegrin University Hospital, 33000 Bordeaux, France; hannah.kaminski@chu-bordeaux.fr; 34 Department of Nephrology, University of Lorrain, CHRU-Nancy, 54511 Vandoeuvre, France; raphaelkormann@gmail.com; 35 CHU Montpellier, Laboratoire de parasitologie-mycologie, Centre national de référence Leishmaniose, Université de Montpellier, UMR MiVEGEC, 34090 Montpellier, France; laurence.lachaud@umontpellier.fr; 36 Service de Néphrologie et Transplantation Rénale, Hôpital Kremlin Bicêtre, 94270 Le Kremlin-Bicêtre, France; christophe.legendre@aphp.fr; 37 Department of Nephrology, Université de Montpellier, CHU de Montpellier, 34090 Montpellier, France; m-lequintrec-donnette@chu-montpellier.fr; 38 INSERM U1285, CNRS UMR 8576, Glycobiology in Fungal Pathogenesis and Clinical Applications, Université de Lille, 59000 Lille, France; jordan.leroy@chru-lille.fr (J.L.); boualem.sendid@univ-lille.fr (B.S.); 39 Pôle de Biologie—Pathologie—Génétique, Institut de Microbiologie, Service de Parasitologie Mycologie, CHU Lille, 59000 Lille, France; 40 Department of Transplantation, Nephrology and Clinical Immunology, Edouard Herriot University Hospital, 69003 Lyon, France; charlene.levi@chu-lyon.fr; 41 Laboratory of Parasitology-Mycology, University of Lorrain, 54511 Nancy, France; m.machouart@chu-nancy.fr; 42 Department of Nephrology and Transplantation, Strasbourg University Hospital, 67000 Strasbourg, France; david.marx@chru-strasbourg.fr; 43 Hospices Civils de Lyon, Institut des Agents Infectieux, Service de Parasitologie et Mycologie Médicale, Hôpital de la Croix-Rousse, 69004 Lyon, France; jean.menotti@univ-lyon1.fr; 44 Institut pour la Recherche et pour le Développement, Microbes, Evolution, Phylogénie et Infection, Institut Hospitalo Universitaire-Méditerranée Infection, Hôpital Conception, Centre de Néphrologie et Transplantation Rénale, Assistance Publique Hôpitaux de Marseille, Aix Marseille Université, 13005 Marseille, France; valerie.moal@ap-hm.fr; 45 Cibles et Médicaments des Infections et de L’immunité, IICiMed, UR1155, CHU de Nantes, Nantes Université, 44000 Nantes, France; florent.morio@chu-nantes.fr; 46 Service de Néphrologie Réanimation Médicale, Pole REUNNIRH, 63000 Clermont-Ferrand, France; nmrozek@chu-clermontferrand.fr; 47 Laboratoire de Microbiologie, Centre Hospitalier Universitaire de Pointe-à-Pitre, Pointe-à-Pitre, 97189 Guadeloupe, France; muriel.nicolas@chu-guadeloupe.fr; 48 Service de Parasitologie-Mycologie, CHU Clermont-Ferrand, 3iHP, INSERM, Université Clermont Auvergne, 63000 Clermont Ferrand, France; ppoirier@chu-clermontferrand.fr; 49 Department of Nephrology, Hôpital Saint-Louis, Assistance Publique Hôpitaux de Paris, 75010 Paris, France; marie-noelle.peraldi@aphp.fr; 50 Centre Hospitalier Bordeaux, Department of Radiology, 33400 Bordeaux, France; bpoussot@hotmail.fr; 51 Assistance Publique-Hôpitaux de Marseille, Institut Hospitalo-Universitaire Méditerranée Infection, Institut de Recherche Pour le Développement, Aix-Marseille Université, Service de Santé des Armées, VITROME, 19-21 Boulevard Jean-Moulin, 13005 Marseille, France; stephane.ranque@univ-amu.fr; 52 Department of Nephrology and Kidney Transplantation, CHU de Limoges, 87000 Limoges, France; jean-philippe.rerolle@chu-limoges.fr; 53 Service de Néphrologie—Transplantation Rénale, Hôpital Foch, 92150 Suresnes, France; renaud.snanoudj@aphp.fr; 54 AP-HP, Service de Néphrologie, Groupe Hospitalier Pitié Salpétrière Charles Foix, 75013 Paris, France; jerome.tourret@aphp.fr; 55 Service de Biologie Clinique and UMR-S 1176, Hôpital Foch, 92150 Suresnes, France; m.vasse@hopital-foch.org; 56 Department of Nephrology, Centre Hospitalier Universitaire Pontchaillou, 35000 Rennes, France; cecile.vigneau@chu-rennes.fr; 57 CHU Strasbourg, Laboratoire de Parasitologie-Mycologie, 67000 Strasbourg, France; odile.villard@chru-strasbourg.fr

**Keywords:** cryptococcosis, opportunistic infection, transplant associated diseases, renal transplantation, cryptococcal meningitis, graft outcome

## Abstract

Cryptococcosis is the third most common cause of invasive fungal infection in solid organ transplant recipients and cryptococcal meningitis (CM) its main clinical presentation. CM outcomes, as well as its clinical features and radiological characteristics, have not yet been considered on a large scale in the context of kidney transplantation (KT). We performed a nationwide retrospective study of adult patients diagnosed with cryptococcosis after KT between 2002 and 2020 across 30 clinical centers in France. We sought to describe overall and graft survival based on whether KT patients with cryptococcosis developed CM or not. Clinical indicators of CNS involvement and brain radiological characteristics were assessed. Eighty-eight cases of cryptococcosis were diagnosed during the study period, with 61 (69.3%) cases of CM. Mortality was high (32.8%) at 12 months (M12) but not significantly different whether or not patients presented with CM. Baseline hyponatremia and at least one neurological symptom were independently associated with CM (*p* < 0.001). Positive serum cryptococcal antigen at diagnosis was also significantly associated with CM (*p* < 0.001). On magnetic resonance imaging (MRI), three patterns of brain injury were identified: parenchymal, meningeal, and vascular lesions. Although CM does not affect graft function directly, it entails a grim prognosis.

## 1. Introduction

*Cryptococcus neoformans* is an opportunistic pathogen ranking as the third most common cause of invasive fungal infection among solid organ transplant recipients [1]. The most frequent type of cryptococcosis manifestation is cryptococcal meningitis (CM), with a prevalence ranging from 25% to 72% across several studies in solid organ transplant recipients [2,3,4]. Taken as a whole, cryptococcosis indicates a grim prognosis with a mortality rate of about 10–25%, while patients with CM fare even worse, with an associated mortality of almost 40% [2,3,4]. The lack of specific symptoms and distinct clinical presentations, as well as an insidious onset, make diagnosis challenging, with significant delays in therapeutic management as a consequence.

Similar to invasive candidiasis—the most prevalent invasive mycosis in immunocompromised hosts—in that it affects both CNS and kidney function, cryptococcosis has not been in the scope of numerous studies [1,5,6]. For CM, studies dedicated to non-human immunodeficiency virus (HIV) patients have further highlighted some of the specific aspects of cryptococcosis in this population, including risk factors associated with neurological involvement [7], the role of plasma and/or cerebrospinal fluid (CSF) cryptococcal antigen (Ag) for prognostic evaluation [8], and the neurological complications of the disease [9]. However, CM outcomes—as well as its clinical features, radiological characteristics, and laboratory findings—have not been analyzed in a large cohort in this particular population [10,11,12].

We therefore conducted a multicentric retrospective study of a cohort of patients with cryptococcosis in 30 kidney transplantation (KT) centers in France.

Our primary objective was to describe the survival of patients with CM as compared to non-CM. Secondary objectives were the description of the radiological characteristics of CM in this specific population, with the aim of defining brain injury patterns and establishing their clinical correlations. We also sought to identify the clinical determinants of CM in KT recipients. Finally, we compared kidney graft survival in the group of patients with CM to patients with cryptococcosis without CM.

## 2. Materials and Methods

### 2.1. Design

This was a retrospective cohort study of adults diagnosed with cryptococcosis after KT between 2002 and 2020 in 30 French transplant centers.

### 2.2. Definitions

Proven CM was defined in compliance with the European Organization for Research and Treatment in Cancer and Mycoses Study Group (EORTC/MSG) definitions [13]: positive culture or ink smear of *Cryptococcus* from CSF or positive Ag in the CSF, or positive histopathological findings in brain tissue yielding 5–10 μm encapsulated yeasts. Patients with cryptococcosis not involving the central nervous system (CNS) were referred to as non-CM cryptococcosis. When lumbar punctures or anatomopathological tissues were not documented, the patient was considered non-CM. Hyponatremia was defined by a plasma sodium level below 135 mmol/L. Mortality rates and graft failure were evaluated 12 months after the diagnosis of cryptococcosis and analyzed throughout the follow-up.

Brain radiological abnormalities were defined as follows: (i) parenchymal lesions: lesions seated in the CNS generating inflammatory hypersignals in fluid-attenuated inversion recovery (FLAIR) imaging or associated with parenchymal enhancement on T1-weighted contrast, or the presence of hematoma; (ii) acute vascular lesions: all lesions displaying signs of acute ischemia on diffusion-weighted imaging and FLAIR imaging; (iii) meningeal lesions: all meningeal enhancement or ventriculitis on T1-weighted contrast; iv) hydrocephalus: increases in the volume of CSF and thus of the cerebral ventricles (ventriculomegaly) based on morphological MR sequences such as T2-weighted imaging.

### 2.3. Inclusion Criteria

Patients were included if they met the following study criteria: KT recipient (single or combined organ transplant) and proven diagnosis of cryptococcosis established between January 2002 and June 2020. Patients were excluded if they were under 18 years or had been on dialysis for more than three months before the diagnosis of cryptococcosis.

### 2.4. Clinical Symptoms and Laboratory Studies

Recipient characteristics, transplant characteristics, immunological parameters, and clinical and biological signs were included using a standardized case collection form. Brain magnetic resonance imaging (MRI) or computed tomography (CT) examinations were performed at the discretion of the physicians.

### 2.5. Statistical Analysis

For the description of continuous variables, the mean and the standard deviation (SD)—whenever they displayed a normal distribution—were used; otherwise, the median and interquartile range were used. The comparisons of the means and the proportions were carried out with the student’s *t*-test (or Mann–Whitney U test if appropriate) and the Chi^2^ test (or Fisher’s exact test if appropriate).

Logistical regression was used to compare patients with CM to their controls (cryptococcosis without meningeal involvement). The variables included in the final multivariate model were selected after a first univariate analysis if they had a degree of significance *p* less than or equal to 0.1.

Graft and patient survival were estimated using the Kaplan–Meier method and compared using the log rank test. The duration of follow-up was calculated from the date of the cryptococcosis diagnosis (starting point) and was followed through up to the date of graft failure or patient death, or at the end of the follow-up.

For patients who died with a functioning graft, graft survival was classified at the time of death as a functional graft.

All analyses were performed using R (version 4.0.0, R Foundation for Statistical Computing, Vienna, Austria) and STATA (StataCorp. 2017- Stata Statistical Software: Release 15. College Station, TX, USA). Values of *p* < 0.05 were considered significant, and all tests were two-tailed.

### 2.6. Ethics

This study was recorded in the Comity National Informatique and Liberty registry (registration number 2212716) in compliance with national policy (20 March 2019).

## 3. Results

Ninety-three patients were identified; three were excluded due to graft failure three months before diagnosis, and two were excluded due to an unspecified date of cryptococcosis diagnosis.

A total of 88 cases were included in the final analysis (Flowchart Figure S1). Of the 88 cases of cryptococcosis, 61 (69.3%) met the definition for CM.

### 3.1. General Characteristics of Patients with CM

#### 3.1.1. Clinical Features

Of the 88 cases of cryptococcosis, the CNS ranked as the most common organ involved (*n* = 61, 69.3%). In 21.6% (*n* = 19) of the cases, the CNS was the only site of infection. The second most frequent organ involved was the respiratory tract (*n* = 28, 31.8%), followed by the skin (*n* = 19, 21.6%). The time between transplantation and cryptococcosis onset did not differ according to the presence or absence of CM (median 34.6 interquartile range (IQR) [1.6–72.4] vs. 42.9 [14.9–121] months, respectively (*p* = 0.581)). Regarding clinical symptoms (see details in Table 1b), patients with CM experienced more frequent vomiting (*p* < 0.001). Conversely, cough or dyspnea were less frequently reported (*p* = 0.049). Headaches (58.2%) and focal neurological signs (34.6%) were the most prevalent neurological signs in CM patients. Fever, present in 58% of cases, was found in a similar proportion whether in CM or non-CM subgroups (*p* = 0.348). Additionally, no difference was recorded when patients with CM were compared to other forms of cryptococcosis for the following conditions: age at diagnosis, post-transplant diabetes, history of other opportunistic fungal infections before cryptococcosis, positive HIV serostatus, induction therapy, or graft rejection before diagnosis (see details in Table 1a). We show a non-significant trend toward a greater prevalence of pre-transplant diabetes in the CM as compared to non-CM *p* = 0.378.

#### 3.1.2. Biological Characteristics

Hyponatremia was common at diagnosis of cryptococcosis, present in 61.8% of all cases (*n* = 42/68). Furthermore, plasma sodium levels were significantly lower in patients with CM, with a median IQR of 132 [130–134] mmol/L vs. 138 [135–139], respectively; *p* < 0.001. Among patients with CM, 25.5% (*n* = 13/51) had CSF white blood cell (WBC) counts within a normal range. CSF protein levels were increased in 76.5% (*n* = 39/51) of CM cases, with a median level of 0.9 [0.6–2.0] g/L. CSF glucose levels were lowered in 65.1% of CM cases (*n* = 28/43). Measurements of intracranial opening pressure were only recorded in 26.2% of CM cases (*n* = 16/61). When performed, pressure was found to be elevated in 93.7% of CM cases, with a median pressure of 30 cm H_2_O [16–32] (Figure S2).

Blood cultures tended to be more frequently positive for cryptococcosis in patients with CM (*p* = 0.055). Upon diagnosis, serum Ag at diagnosis was also more frequently positive in cases of CM (*p* < 0.001, Table 1c). When sampled, CSF antigen at diagnosis was positive in 100% (*n* = 33/33) of CM cases, the India ink test of the CSF yielded positive results for 41.2% (*n* = 21/51) of the patients, and CSF cultures were positive in 31.4% (*n* = 16/51) of cases (Table 1c). Different types of serological tests were used to detect Cryptococcus: enzyme-linked immunosorbent assay (ELISA) in 22% of cases (*n* = 11/50), Latex in 70% (*n* = 35/50), and immunochromatography in 8% (*n* = 4)

#### 3.1.3. Immunosuppressive Regimen

The induction therapy and the immunosuppressive regimen were similar between the patients, irrespective of CM. Additionally, there was no difference in the type of calcineurin inhibitor (tacrolimus or ciclosporin) between patients with or without CM (*p* = 0.246).

#### 3.1.4. Therapeutic Management

Among patients with CM, 77.8% (*n* = 42/54) received liposomal amphotericin B combined with flucytosine, as recommended by current guidelines.

The remaining patients received a single drug therapy or a combination of treatments that were not in line with recommendations. Of the patients who did not receive a treatment in keeping with guidelines, 9.6% (*n* = 5/54) died in the years following treatment, and in 80% (*n* = 4/5) of cases, death was directly ascribable to cryptococcosis (one patient relapsed 9 months after the onset of cryptococcosis; Table S1).

A total of 34.4% (*n* = 21/61) of patients required iterative lumbar punctures to manage intracranial hypertension. Seven patients required neurosurgical intervention for the placement of an external ventricular drain for the management of intracranial hypertension (11.5%, *n* = 7/61).

#### 3.1.5. Determinants of CM

The determinants of CM were investigated based on the univariate analysis detailed in Table 2.

Upon logistic regression analysis, the determinants independently associated with CM included the presence of at least one neurological symptom with an odds ratio (OR) of 60.71 (95% CI [9.12 to 404.20], *p* < 0.001) and natremia at the time of diagnosis (per 1 mmol/L increment) with an OR of 0.76 (95% CI [0.63 to 0.93], *p* = 0.008), as detailed in Table 3.

### 3.2. Prognosis of CM vs. Non-CM Cryptococcosis

#### 3.2.1. Diagnostic Delay

**Figure 1:**
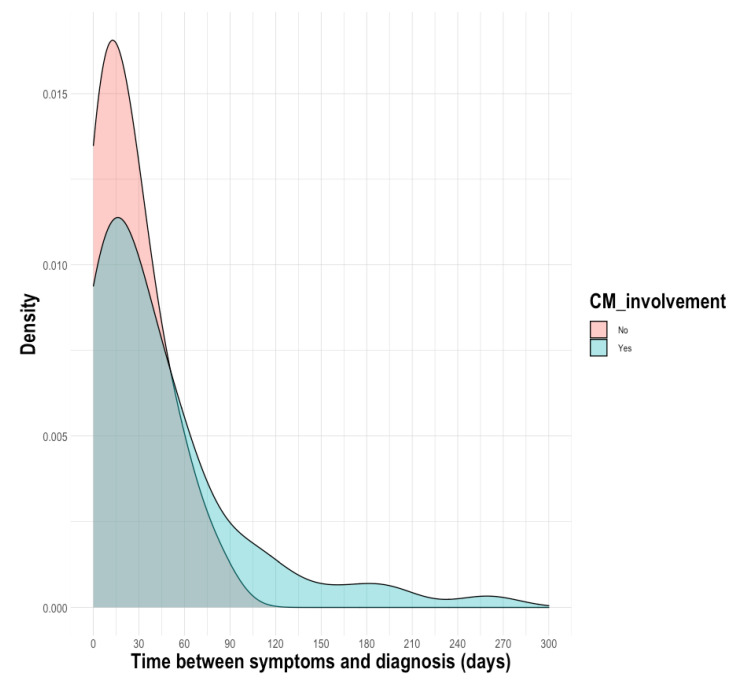
Density plot of distribution of the time between the first symptoms and the diagnosis.

There was a delay between the diagnosis of cryptococcosis and the first symptoms, with a median (IQR) of 23 days (7–52) for patients with cryptococcosis. Compared to patients with CM, the time interval did not differ in the subgroup of patients with non-CM, with a median of 25 days (9–58) vs. 21 days (5–34), *p* = 0.0823 (Figure 1). However, as shown on the density plot, some patients were diagnosed very late after the onset of CM (>120 days).

#### 3.2.2. Patient Outcomes

At 12 months post-diagnosis, 32.8% of patients with CM died compared to 25.9% in the non-CM group (Table 4).

**Figure 2:**
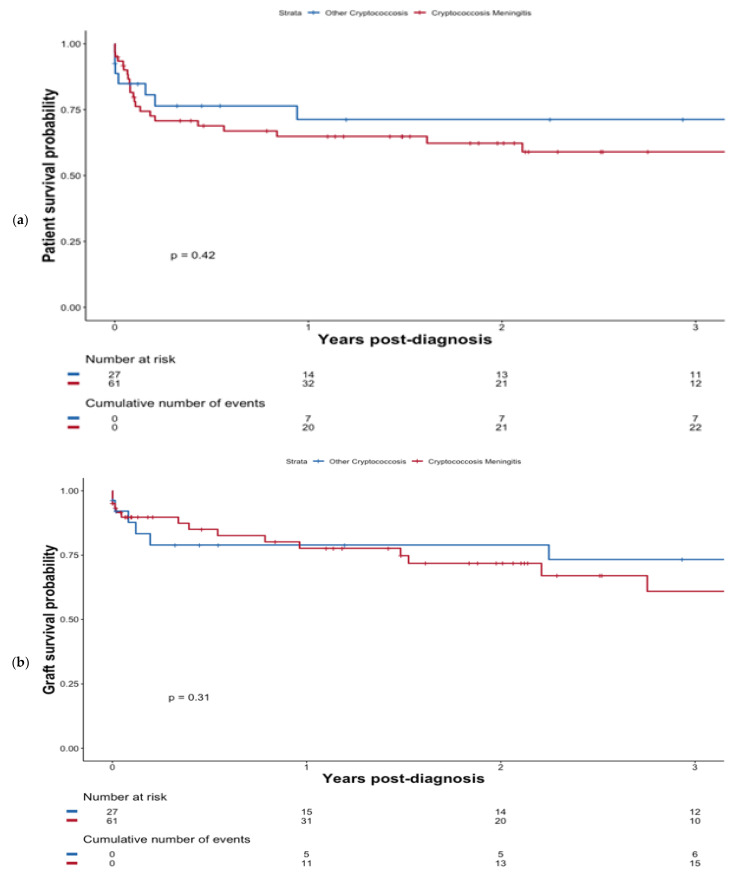
Patient survival probability (**a**) and graft survival probability (**b**) after diagnosis of cryptococcosis depending on whether patients presented with CM or not.

At last follow-up, a total of 26 (42.6%) of patients with CM died at a median of 1.2 months (0.8–10.0) following diagnosis. However, CM did not confer worse patient survival compared with non-CM patients, (*p* = 0.42); see Figure 2a. Additionally, the presence of fungemia did not significantly impact survival (*p* = 0.27; Figure S3). Overall, six patients developed an immune reconstitution inflammatory syndrome (IRIS), including 83.3% (*n* = 5/6) in the CM group.

#### 3.2.3. Graft Survival

At the last follow-up, a total of 19 (31.1%) patients with CM experienced graft failure at a median of 9.4 months (0.2–33.0) post-diagnosis. Graft survival was not significantly altered in the case of CM (*p* = 0.31) see Figure 2b.

### 3.3. CM-Associated Features in Brain Imaging

#### 3.3.1. Radiological Characteristics

Brain imaging (MRI and/or CT) was performed in 96.7% (*n* = 59/61) of cases with CM, using MRI (*n* = 41, 71.9%) or CT (*n* = 12, 21.0%) exclusively. When performed, CT scans did not yield positive findings, even in cases of confirmed CM, except for hydrocephalus (*n* = 1). There were significantly more abnormal MRIs than CT scans in the case of CM (*p* = 0.004). These results are summarized in Figure S4.

**Figure 3:**
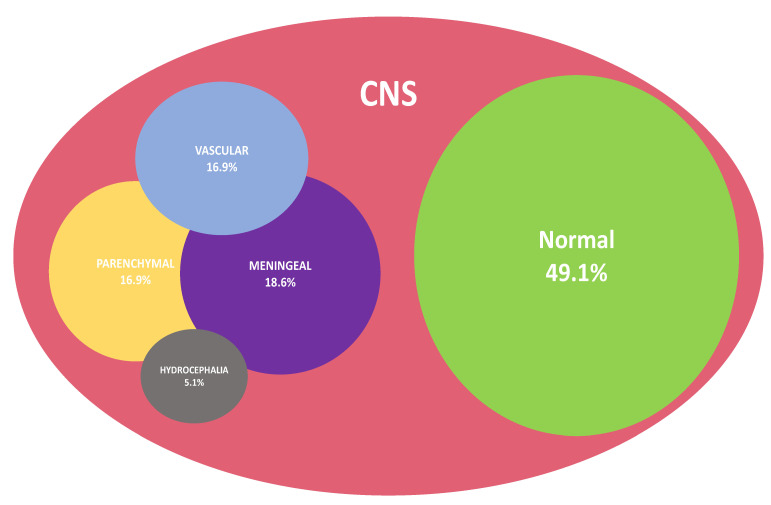
Venn diagram representation summarizing brain injury patterns as displayed in imaging of patients with CM (*n* = 59/61).

When performed, measurements of intracranial opening pressure were found to be elevated in 93.7% of CM cases. On brain imaging, the predominant injury patterns consisted of meningeal damage (*n* = 11, 18.6%), followed by parenchymal lesions (*n* = 10, 16.9%) and vascular injury (*n* = 10, 16.9%). Patients could display a combination of these patterns, as detailed in Figure 3. Cases of subtypes of brain lesions are shown in Figure S5.

#### 3.3.2. Clinical Presentation According to Brain Injury Pattern

Symptoms did not differ depending on whether or not patients disclosed vascular injury in imaging. More specifically, patients with focal neurological signs were not found to be more frequent among patients with vascular injury on imaging; *p* = 0.256 (Table S2).

The pattern of symptoms was similar regardless of whether patients exhibited hydrocephalus in imaging. Specifically, patients with hydrocephalus did not have more headaches (*p* = 0.599; Table S3).

#### 3.3.3. Outcomes According to the Type of Radiological Characteristics

**Figure 4:**
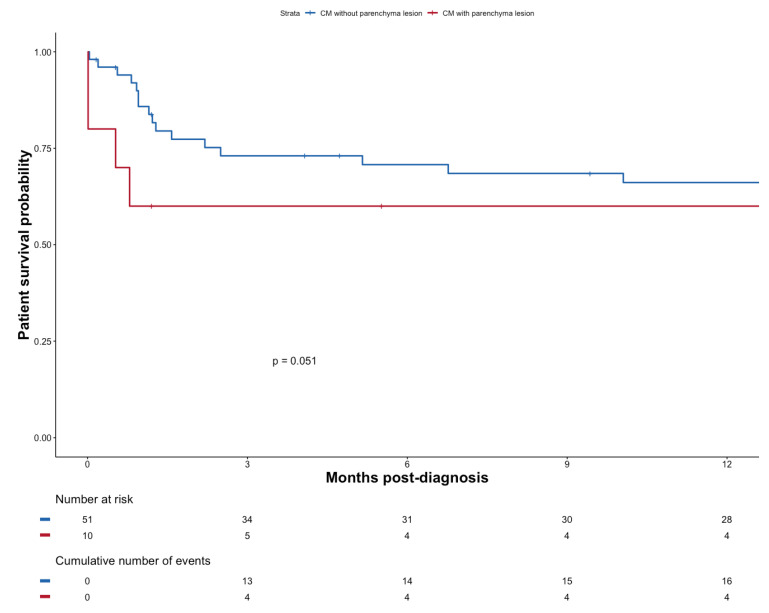
Patient survival probability after diagnosis of CM with or without parenchyma lesion.

Mortality tended to be worse if patients exhibited signs of brain parenchymal injury (*n* = 10); *p* = 0.051 (Figure 4).

**Figure 5:**
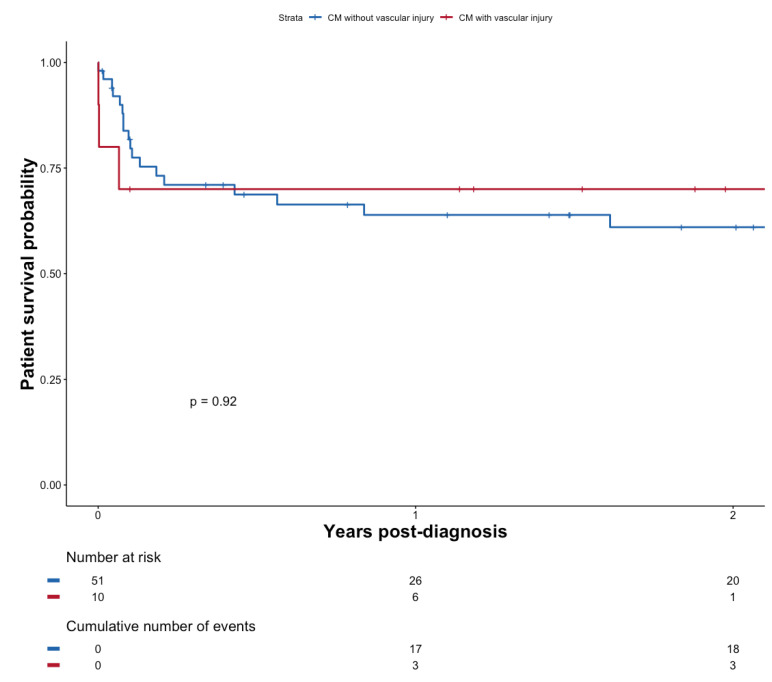
Patient survival probability after diagnosis of CM with or without vascular injury.

There was no significant survival difference after diagnosis of CM, whether patients presented evidence of vascular injury on the brain MRI (*n* = 10) or not (*n* = 49), *p* = 0.92 (see Figure 5). Evidence of vascular injury in imaging was not associated with reduced diagnostic delay (*p* = 0.9754).

## 4. Discussion

### 4.1. Clinical Features and Risk Factors

Neurotropism is one of the most prominent clinical features of pathogenic species of the genus *Cryptococcus*, explaining why CM is by far the most frequently identified clinical condition in cryptococcosis. Susceptibility to CNS infection is a universal trait of cryptococcosis, regardless of the underlying mechanism of immunosuppression (IS), but has been found to be even more pronounced in non-HIV-related IS compared to HIV-infected patients [7,8,9,14]. The clinical pattern of cryptococcosis-related CNS disease encompasses a broad range of neurological symptoms ranging from headaches to seizures. In our study, the presence of at least one neurological symptom was independently associated with CM. However, the predominant clinical picture is inconspicuous; in our study, a significant segment of our study population presented with non-specific signs (headaches, emesis, or dizziness), the neurological origin of which may easily have been discounted.

This is in line with previous studies in HIV-negative individuals where headaches appeared to be the leading manifestation, although with varying rates (24% to 100%) [15]. Bearing in mind that fever was only detected in 58% of patients, this makes for a misleading clinical picture that translates into significantly delayed diagnoses, which in turn exposes the patients to a heightened risk of mortality and debilitating neurological sequalae [9,14]. As a result, our patients with CM tend to experience prolonged diagnostic delays, compared to patients with non-CM cryptococcosis.

Consistent with previous studies, hyponatremia [16] was closely associated with the occurrence of CM. The underlying mechanism connecting hyponatremia with CM is unclear. Emesis and hyponatremia may well be interconnected, considering the pathophysiological interplay of emesis, hypovolemia, and arginine vasopressin release [16,17]. Nevertheless, clinicians should be alert to meaningful cues such as hyponatremia, together with a history of headaches and emesis.

Our study shows that a significant proportion of patients with proven CM display a normal count of WBC cells in the CSF (19.7%), a result akin to HIV patients with CM but hitherto unrecognized in transplant patients [18,19]. The urgent need for a timely diagnosis in the face of indefinite neurological-like symptoms, together with an unremarkable CSF examination, has spurred investigators to develop alternative strategies. The use of serum antigen is one such option.

Previous studies have shown that high titers of serum AG titer (>1/64) can accurately predict CM whenever patients present with clinical neurological manifestations [20,21]. Unfortunately, in our study, the serum AG titer could not be analyzed specifically due to an inordinately high level of missing data. However, in any case, serum testing is no substitute for clinician awareness: the success of this strategy presupposes increased vigilance and broad indications for serum AG titer testing.

In addition, high levels of serum AG titer do not obviate the need for the lumbar puncture required to document an elevated opening pressure (>25 mm Hg). This was demonstrated in a significant subset of patients in this study, higher than in other non-specific KT reports (22% [2] to 44% [14]).

Elevated opening pressure reflects raised intracranial pressure and carries its own implications in terms of survival, requiring repeat lumbar punctures or the placement of a permanent shunt [14]. It is thus meaningful that opening pressure was only documented in a fraction (26.5%) of the patient population—a deviation from current guidelines which has been reported in other settings. In our study, elevated intracranial pressure was detected in 83.3% of cases. Taken together, these results suggest further cases of intracranial hypertension may have been detected had lumbar punctures been performed more widely.

### 4.2. Outcome

In this cohort, cryptococcosis-associated mortality did not differ whether patients presented with CM or not at 12 months or at the last follow up (42% vs. 37%, *p* = 0.798). The fact that lumbar puncture was not carried out in a consistent fashion in our cohort may have obscured the significance of our results. Compared to the existing literature, the overall mortality when merging the CM and non-CM cryptococcosis subgroups was found to be higher. For instance, a large previous study, conducted in solid organ transplant patients, showed a mortality rate ranging between 15 and 20%—with the greatest rate (20%) observed in cases of CM [10].

Nevertheless, whenever KT patients are considered specifically, mortality rates have been shown to be comparable to our results; a recent study reported a mortality of 41% at 12-months follow-up in this subgroup [3]. Similarly, a series of 29 patients with cryptococcosis yielded a mortality rate of 34% at the last follow up [22]. Graft outcomes in our study were not significantly different for cases of CM.

### 4.3. Characteristics Based on CNS Imaging

According to our series, the pattern of injury of the CNS could be classified into three broad categories based on imaging—namely, vascular damage (*n* = 10), parenchymal lesions (*n* = 10) and meningeal (pachymeningeal and leptomeningeal) lesions (*n* = 11), with a near-even distribution.

Prior studies have produced conflicting results with respect to the type of CNS lesion upon imaging. In one study focusing on organ transplant recipients [10], cerebrovascular lesions were not described. Conversely, in recent reports [18,23] that included HIV negative patients, cerebral ischemic lesions were detected in 15–43% of these patients and their incidence was significantly higher in this segment of the population compared to HIV-positive patients. One explanation for these contrasting rates is partly due to the fact that Virchow–Robin spaces engulfed with fungus may closely mimic infarcts on diffusion restriction MRI, giving rise to differing interpretations. A recent retrospective analysis of 66 patients meeting the criteria for CM and investigated with appropriate brain imaging identified 20 patients (30.3%) with cerebral infarcts—all of which were of the lacunar type and often manifold (50%). Based on neuropathological reports and other forms of chronic meningitis recognized to involve vasculitis—first and foremost being tuberculous meningitis [24]—cerebrovascular injury is believed to arise through (i) dissemination of the inflammatory process to the vessels, originating either from basal exudates or meningeal infection and causing vascular strangulation and thrombosis; (ii) raised intracranial pressure hindering cerebral blood flow.

In our work, 38.7% of the patients with CM exhibited cerebrovascular involvement. In this subgroup of patients, symptoms related to an acute or sub-acute stroke may be partly veiled by other clinical features related to cryptococcosis and therefore go unrecognized. In keeping with this interpretation, clinical features did not differ whether brain imaging yielded evidence of vascular injury or not (*p* = 0.256). We failed to demonstrate that the type of CNS injury as characterized by imaging exerted a meaningful impact on survival. However, the association between brain parenchymal lesions and mortality verged on significance (*p* = 0.051), although the small cohort size means that the study may have been underpowered to detect any significant difference. Furthermore, the study was not devised to evaluate long-term persistent neurological impairment—a relevant issue given the prevalence of ischemic lesions.

## 5. Conclusions

Akin to other immunocompromised patients, this study confirms that the CNS is the most common organ affected when KT recipients develop cryptococcosis. Patient management is impaired by significantly protracted diagnostic delays. Non-specific symptoms may account for such delays. Our results suggest that clinicians should heed neurological manifestations, headaches, and hyponatremia, which hint at the occurrence of CM. Whenever cryptococcosis is confirmed, these signs should compel clinicians to perform lumbar punctures to both substantiate CM, assess opening pressure and, if need be, to remove CSF to control intracranial pressure. Our results also corroborate the emergence of a novel distinct radiological entity defined by vascular lesions. Meanwhile, patients exhibiting parenchymal lesions seem to be at a higher risk of more severe outcomes. Overall, compared to non-CM cryptococcosis patients, KT patients with CM do not fare worse with respect to patient and graft survival. Worryingly, in the setting of KT, the prognosis of patients with cryptococcosis remains dismal on both counts. In fact, the outcome of KT patients developing CM may be even more severe than in other immunocompromised patients.

## Figures and Tables

**Table 1:** (**a**) Characteristics of CM and non-CM before diagnosis; (**b**) Characteristics of CM and non-CM at diagnosis and follow-up; (**c**) Microbiological characteristics of CM and non-CM at diagnosis.

(a)			
Characteristics	*Non-CM n = 27*	*CM n = 61*	*p* Value
Clinical characteristics			
Male, No. (%)	21 (77.8)	40 (65.6)	0.371
Age at diagnosis (years), mean (SD)	58.07 (12.8)	57.23 (13.6)	0.786
BMI (kg/m^2^), mean (SD)	22.9 (3.28)	23.7 (5.94)	0.472
Pre-transplant diabetes, No. (%)	3 (11.1)	12 (19.7)	0.378
Post-transplant diabetes, No. (%)	12 (44.4)	28 (45.9)	1.000
HIV status, No. (%)	1 (3.7)	3 (4.9)	1.000
Baseline eGFR before cryptococcosis (CKD-EPI in ml/min/1.73 m^2^), mean (SD)	46.5 (20.4)	48.0 (27.8)	0.779
RRT duration (months), median [IQR]	48 [19–108]	32 [12–50]	0.195
Prior transplant, No. (%)	5 (20)	12 (19.7)	0.972
Deceased donor type (vs. living), No. (%)	24 (88.9)	57 (93.4)	0.671
Length of post-transplant hospitalization (days), mean (SD)	20.4 (13.2)	19.0 (10.6)	0.706
Positive Anti-HLA donor specific antibody at time of transplant, No. (%)	6 (26.1)	12 (24.5)	1.000
Immunosuppressive induction			
None, No. (%)	2 (9.5)	1 (2.0)	0.222
ATG, No. (%)	14 (66.7)	36 (70.6)	0.642
IL2R, No. (%)	5 (23.8)	14 (27.5)	0.782
Other infections prior cryptococcosis			
Infections, No. (%)			
Fungal infections	3 (11.1)	2 (3.28)	0.170
CMV disease	1 (4)	9 (15.2)	0.268
PCR BK positive during follow-up	2 (12.5)	3 (8.1)	0.632
**(b)**			
**Characteristics**	***Non-CM n = 27***	***CM *n* = 61***	***p* value**
Time between transplantation and diagnosis (months), median [IQR]	42.9 [14.9; 121]	34.6 [11.6; 72.4]	0.581
Time between first symptoms and diagnosis (days), median [IQR]	21 [5–34]	24 [9–58]	0.112
Admission delay—diagnosis (days), median [IQR]	5 [2–11]	8 [2–18]	0.466
Number of hospitalizations/consultations with functional complaint before hospitalization, median [IQR]	1 [0–2]	1 [0–2]	0.338
Total length of hospitalization (days), median [IQR]	16 [8–47]	30 [21–43]	0.042
ICU admission, No. (%)	6 (25)	25 (42.4)	0.210
Need for mechanical ventilation, No. (%)	0	17 (27.9)	0.001
ICU duration (days), median [IQR]	4.5 [1–8]	4 [1–13]	0.816
Clinical presentation at diagnosis, No. (%)			
Asthenia	9 (34.6)	27 (45.0)	0.477
Emesis	0	21 (35.0)	<0.001
Digestive disorder	4 (15.4)	8 (13.3)	0.749
Skin lesion	5 (19.2)	4 (6.7)	0.122
Cough/dyspnea	9 (34.6)	9 (15)	0.049
Fever at diagnostic	13 (50)	37 (61.7)	0.348
Natremia at diagnosis (mmol/L), median [IQR]	138 [135–139]	132 [130–134]	<0.001
Hyponatremia at diagnosis (<135 mmol/L), No. (%)	3 (15.8)	39 (78.0)	0.005
Total lymphocytes at diagnosis (mm^3^), median [IQR]	960 [340–2000]	755 [500–1200]	0.600
CD4 count at diagnosis (mm^3^), median [IQR]	79 [51–118]	89 [46–202]	0.754
Intracranial pressure at diagnosis(cmH_2_0), median [IQR]	17.5 [12–23]	29 [15.5–31]	0.258
Co-infection associated with diagnosis (viral, bacterial or fungal), No. (%)	10 (43.5)	20 (34.5)	0.457
eGFR (CKD-EPI in ml/min/1.73 m^2^) at diagnosis (*n* = 74), mean (SD)	38.6 (17.1)	39.9 (21.0)	0.806
**(c)**			
**Characteristics**	***Non-CM n = 27***	***CM n = 61***	***p* value**
Cryptococcus species *, No. (%)			1.00
Cryptoccocus *deneoformans* (ex C. *neoformans*)	22 (88)	49 (84.5)	
Cryptococcus *neoformans* *(ex C.* *grubii*)	3 (12.0)	9 (15.5)	
Positive serum Ag at diagnosis (*n* = 73)	11 (20.0)	44 (80.0)	<0.001
Serum Ag titer at diagnosis median [IQR] (*n* = 29)	1/160 [1/4–1/2018]	1/114 [1/20–1/512]	0.935
Positive serum cultures at diagnosis	6 (24.0)	29 (47.5)	0.055
Positive CSF Ag at diagnosis No. (%)	-	33 (100)	
Positive India ink test CSF, No. (%)	-	21 (40.4)	
CSF Ag titer at diagnosis, median [IQR]	-	1/64 [1/8–1/450]	
Positive culture in CSF, No. (%)	-	16 (29.1)	
CSF WBC count (/mm^3^), median [IQR]	2.5 [2–3.5]	55 [5–175]	0.001

NOTE. Ag: cryptococcal antigen; ATG: antithymocyte globulin; BK: BK virus; BMI: body mass index; CD4: CD4 T lymphocyte cells; CKD-EPI: Chronic Kidney Disease Epidemiology; CM: cryptococcal meningitis; CMV: cytomegalovirus; CSF: cerebrospinal fluid; diagnosis: date of the diagnosis of cryptococcosis; eGFR: estimated glomerular filtration rate; HIV: human immunodeficiency virus; HLA: human leukocyte antigen; ICU: intensive care unit; IL2R: interleukin 2—receptor; IQR: interquartile range; PCR: polymerase chain reaction; RRT: renal replacement therapy; SD: standard deviation; WBC: white blood cells.* C. *gattii* was not detected in our cohort.

**Table 2:** Determinants of CM in the univariate analysis.

Characteristics	*N*	OR [95% CI]	*p* Value
**Clinical characteristics**			
**Age at time of diagnosis** (per 1 year increment)	88	0.99 [0.96;1.03]	0.783
**HIV status**	88		
Negative		Ref.	
Positive		1.34 [0.13;13.5]	0.797
**Number of cardiovascular risk factors, (per 1 risk factor increment)**	84	1.48 [0.93;2.36]	0.088
**Graft rejection before cryptococcosis**	87		
No		Ref.	
Yes		0.44 [0.17;1.17]	0.102
**Tacrolimus at time of diagnosis**	83		
No		Ref	
Yes		1.77 [0.64;4.87]	0.275
**eGFR (CKD-EPI) at time of diagnosis (per 1 mL/min/1.73 m^2^ increment)**	85	1.01 [0.99;1.03]	0.504
**Time between transplant and diagnosis (per 1 year increment)**	79	1.54 [0.33;7.22]	0.533
**Clinical presentation**			
**ICU hospitalization:**	83		
No		Ref.	
Yes		2.21 [0.77;6.36]	0.131
**Natremia at time of diagnosis (per 1 mmol/l increment)**	**69**	**0.71 [0.59;0.85]**	**<0.001**
**At least one neurological symptom at time of diagnosis**	86		
No		Ref.	
**Yes**		**90.85 [17.56;470.16]**	**<0.0001**
**Fever at time of diagnosis**	84		
No		Ref	
Yes		1.69 [0.66;4.35]	0.272
**Skin lesion**	86		
No		Ref.	
Yes		0.31 [0.07;1.23]	0.094
**Cough/Dyspnea**	86		
No		Ref.	
Yes		0.33 [0.11;0.98]	0.046
**Fungemia**	86		
No		Ref.	
Yes		2.87 [1.01;8.17]	0.041

NOTE. CKD-EPI: Chronic Kidney Disease Epidemiology; eGFR: estimated glomerular filtration rate; HIV: human immunodeficiency virus; ICU: intensive care unit; OR: odds ratio. Fungemia refers to bloodstream infection caused by *Cryptococcus sp.*

**Table 3:** Determinants of CM in the multivariate analysis.

	*N*	OR [95% CI]	*p* Value
Natremia at time of diagnosis (per 1 mmol/L increment)	69	0.76 [0.63;0.93]	0.008
At least one neurological symptom at time of diagnosis	69		
No		Ref.	
Yes		60.71 [9.12;404.20]	<0.001

**Table 4:** Outcomes after cryptococcosis comparing CM and non-CM.

Characteristics	Non-CM *n* = 27 (%)	CM *n* = 61 (%)	*p* Value
IRIS, No (%)	1 (3.70)	5 (8.20)	0.662
eGFR M12 after cryptococcosis (CKD-EPI), mean (SD)	48.8 (32.4)	35.9 (15.4)	0.209
eGFR at last follow-up (CKD-EPI), mean (SD)	40.1 (18.7)	35.7 (20.7)	0.430
Graft failure at last follow-up, No (%)	9 (34.6)	19 (38)	0.808
Patient death at M12, No (%)	7 (25.9)	20 (32.8)	0.594
Patient death at last follow-up, No (%)	10 (37.0)	26 (42.6)	0.798

NOTE. CKD-EPI: Chronic Kidney Disease Epidemiology; eGFR: estimated glomerular filtration rate; HIV: human immunodeficiency virus; ICU: intensive care unit; IRIS: immune reconstitution inflammatory syndrome; SD: standard deviation.

## Data Availability

The data and material are available upon request to the corresponding author.

## Supplementary Materials

The following supporting information can be downloaded at: https://www.mdpi.com/article/10.3390/pathogens11060699/s1, Figure S1: Flow Chart; Figure S2: Measurement of intracranial pressure in patients with cryptococcosis; Figure S3: Patient survival probability after diagnosis of Cryptococcosis with fungemia; Figure S4: Results of brain imaging by type for CM patients; Figure S5: MRI patient on magnetic resonance imaging; Table S1: Summary table of the clinical evolution of patients with induction treatment outside Guidelines; Table S2: Neurological symptoms based on the detection of vascular injury on brain imaging; Table S3: Neurological symptoms based on the detection of hydrocephalus on brain imaging.
