# Supplementary material for: Cryptococcal Meningitis in Kidney Transplant Recipients: A Two-Decade Cohort Study in France
Source: Pathogens. 2022 Jun 17;11(6):699. doi: 10.3390/pathogens11060699 (PMC9227085; doi:10.3390/pathogens11060699)
Supplement: Supplementary file 1 [file pathogens-11-00699-s001.zip › pathogens-1706778-supplementary/pathogens-1706778-supplementary/Figure S1.pdf]

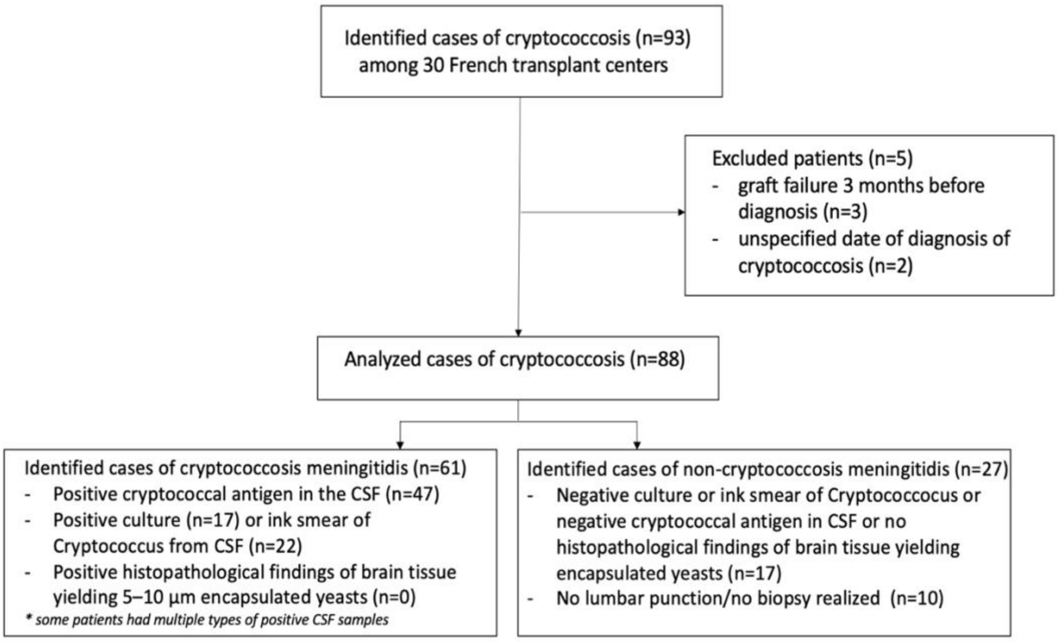

Figure S1. Ninety-three patients were identified, 3 were excluded due to graft failure 3 months before diagnosis and 2 were excluded due to an unspecified date of diagnosis of cryptococcosis. A total of 88 cases were included in the final analysis.
