# Supplementary material for: Cryptococcal Meningitis in Kidney Transplant Recipients: A Two-Decade Cohort Study in France
Source: Pathogens. 2022 Jun 17;11(6):699. doi: 10.3390/pathogens11060699 (PMC9227085; doi:10.3390/pathogens11060699)
Supplement: Supplementary file 1 [file pathogens-11-00699-s001.zip › pathogens-1706778-supplementary/pathogens-1706778-supplementary/Figure S2.pdf]

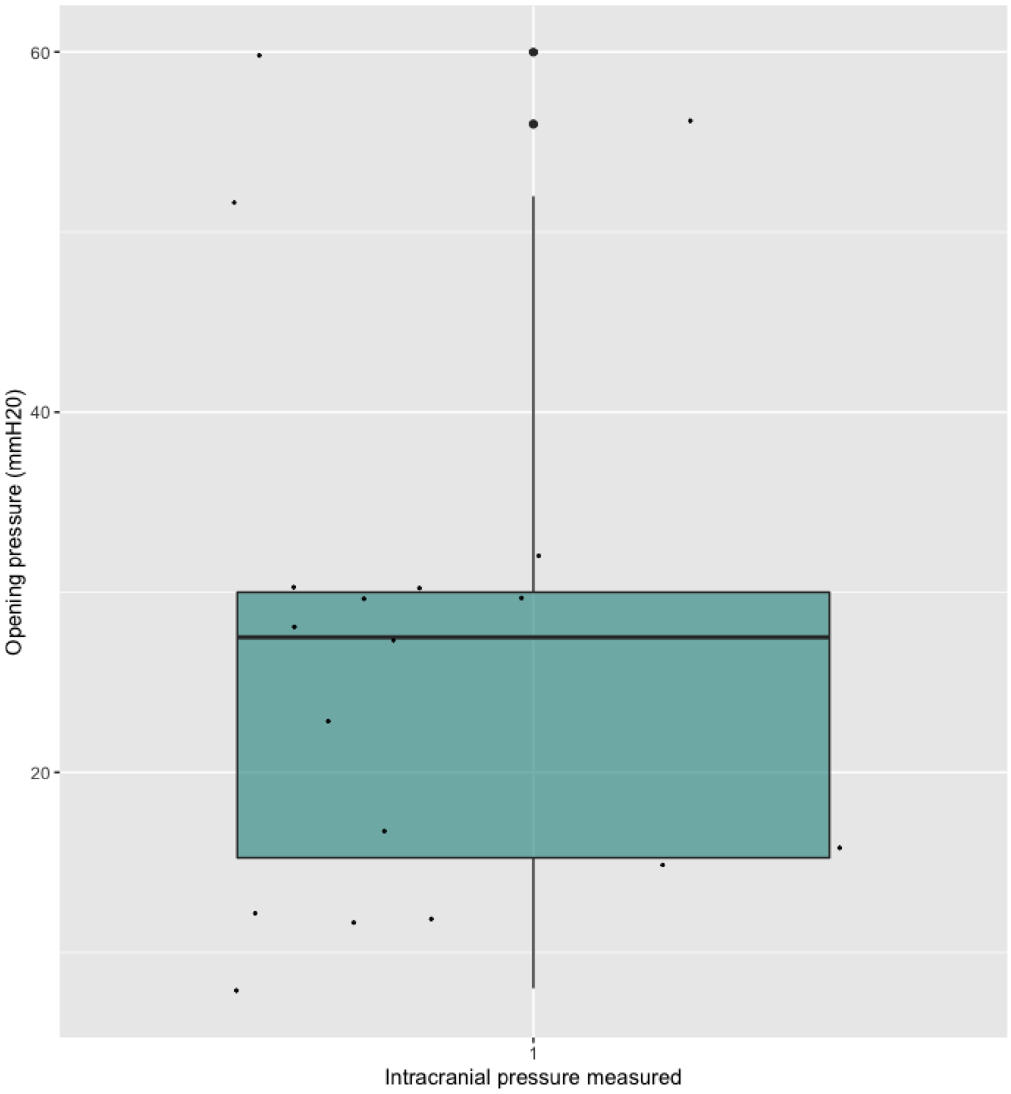

Figure S2. Measurement of intracranial opening pressure was only reported in 26.5% of cases (n = 18/68) and 26.2% of CM cases. When performed, pressure was found to be elevated in 83.3% of cases, with a median pressure of 27.5 cmH20 [15-30]. Regarding patients with CM, pressure was found to be elevated in 93.7% of CM cases, with a median pressure of 30 cmH20 [16-32].
