# Supplementary material for: Cryptococcal Meningitis in Kidney Transplant Recipients: A Two-Decade Cohort Study in France
Source: Pathogens. 2022 Jun 17;11(6):699. doi: 10.3390/pathogens11060699 (PMC9227085; doi:10.3390/pathogens11060699)
Supplement: Supplementary file 1 [file pathogens-11-00699-s001.zip › pathogens-1706778-supplementary/pathogens-1706778-supplementary/Figure S5.pdf]

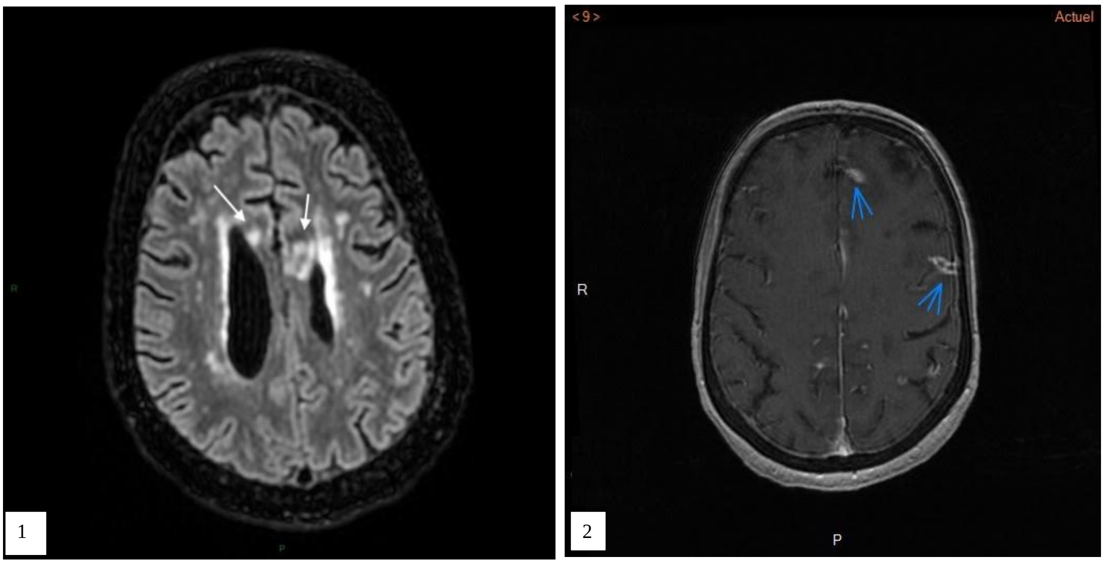

Figure S5. MRI patient on magnetic resonance imaging (MRI)

Patient 1: Patient on MRI including diffusion-weighted imaging (DWI) and fluid-attenuated inversion recovery (FLAIR) showing bilateral multiple strokes located in the corpus callosum (white arrows) along with chronic ischemic small vessel disease. No sign of meningeal or parenchymal abnormalities on gadolinium enhanced sequences.

Patient 2: MRI patient on magnetic resonance imaging (MRI) with gadolinium enhanced T1 sequences showing multiple leptomeningeal enhancement foci (blue arrow) in the left frontal lobe.
