# Supplementary material for: Cryptococcal Meningitis in Kidney Transplant Recipients: A Two-Decade Cohort Study in France
Source: Pathogens. 2022 Jun 17;11(6):699. doi: 10.3390/pathogens11060699 (PMC9227085; doi:10.3390/pathogens11060699)
Supplement: Supplementary file 1 [file pathogens-11-00699-s001.zip › pathogens-1706778-supplementary/pathogens-1706778-supplementary/Table S1.pdf]

Table S1. Summary table of the clinical evolution of patients with induction treatment outside Guidelines

Induction treatment	N	Alive at M12
Fluconazole or Voriconazole	6	2/6
Flucytosine + Voriconazole or Fluconazole	4	3/4
Amphotericine B (liposomale)	2	2/2
