# Supplementary material for: Cryptococcal Meningitis in Kidney Transplant Recipients: A Two-Decade Cohort Study in France
Source: Pathogens. 2022 Jun 17;11(6):699. doi: 10.3390/pathogens11060699 (PMC9227085; doi:10.3390/pathogens11060699)
Supplement: Supplementary file 1 [file pathogens-11-00699-s001.zip › pathogens-1706778-supplementary/pathogens-1706778-supplementary/Table S2.pdf]

Table S2. Neurological symptoms based on the detection of vascular injury on brain imaging

	No Vasculary injury n=49	Vascular injury n=10	p-value
Fever, No (%)	29 (59.2%)	8 (80.0%)	0.294
Headache, No (%)	26 (53.1%)	5 (50.0%)	1.000
Focal neurological signs, No (%)	13 (26.5%)	5 (50.0%)	0.256
Dizziness, No (%)	6 (12.2%)	1 (10.0%)	1.000
Blurred vision, No (%)	4 (8.16%)	2 (20.0%)	0.266
Seizures, No (%)	5 (10.2%)	1 (10.0%)	1.000
Confusion, No (%)	12 (24.5%)	2 (20.0%)	1.000
