# Supplementary material for: Cryptococcal Meningitis in Kidney Transplant Recipients: A Two-Decade Cohort Study in France
Source: Pathogens. 2022 Jun 17;11(6):699. doi: 10.3390/pathogens11060699 (PMC9227085; doi:10.3390/pathogens11060699)
Supplement: Supplementary file 1 [file pathogens-11-00699-s001.zip › pathogens-1706778-supplementary/pathogens-1706778-supplementary/Table S3.pdf]

Table S3. Neurological symptoms based on the detection of hydrocephalus on brain imaging

	**No hydrocephalus n=56**	**Hydrocephalus n=3**	**p-value**
Fever, No (%)	36 (64.3%)	1 (33.3%)	0.549
Headache, No (%)	30 (53.6%)	1 (33.3%)	0.599
Focal neurological signs, No (%)	17 (30.4%)	1 (33.3%)	1.000
Dizziness, No (%)	6 (10.7%)	1 (33.3%)	0.320
Blurred vision, No (%)	6 (10.7%)	0 (0.0%)	1.000
Seizures, No (%)	6 (10.7%)	0 (0.0%)	1.000
Confusion, No (%)	13 (23.2%)	1 (33.3%)	0.564
