# Supplementary figures and images for: Cryptococcal Meningitis in Kidney Transplant Recipients: A Two-Decade Cohort Study in France
Source: Pathogens. 2022 Jun 17;11(6):699. doi: 10.3390/pathogens11060699 (PMC9227085; doi:10.3390/pathogens11060699)

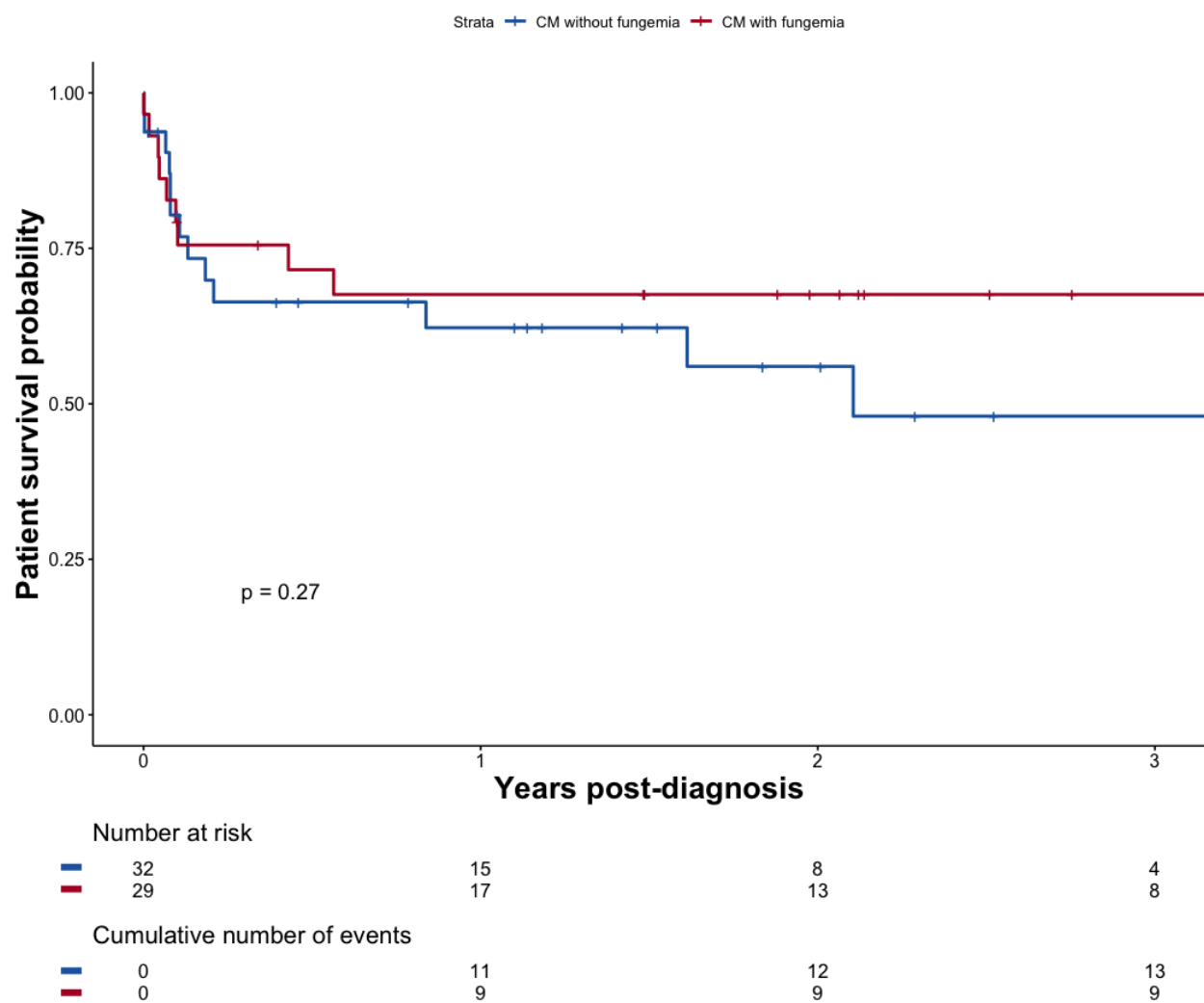

**Figure S3.** Patient survival probability after diagnosis of Cryptococcosis with fungemia.

Supplement: Supplementary file 1 [file pathogens-11-00699-s001.zip › pathogens-1706778-supplementary/pathogens-1706778-supplementary/Figure S3.pdf]

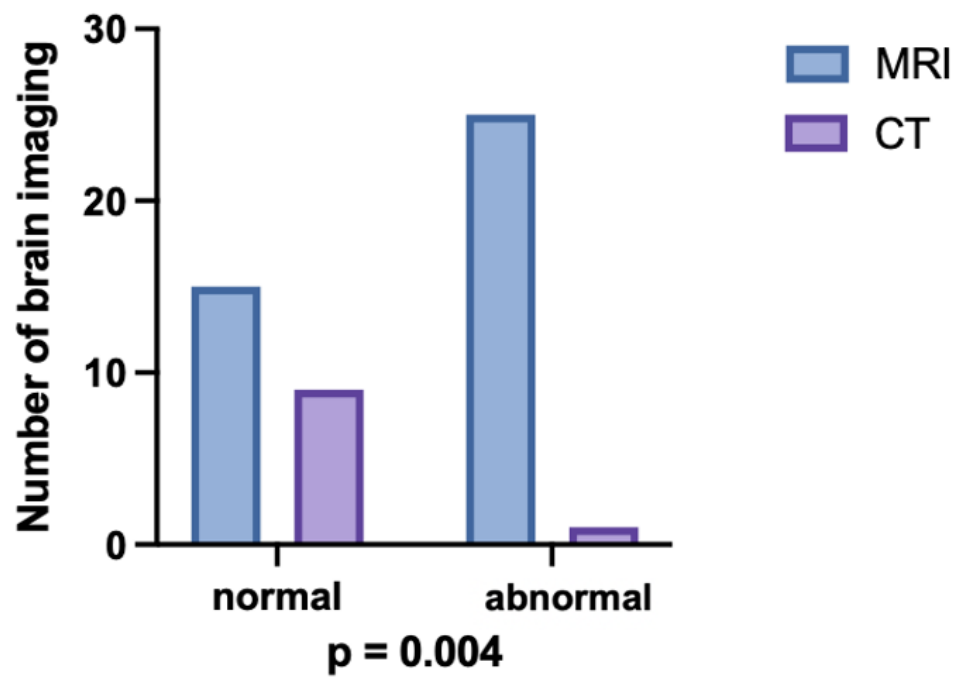

Figure S4. Results of brain imaging by type for CM patients

Supplement: Supplementary file 1 [file pathogens-11-00699-s001.zip › pathogens-1706778-supplementary/pathogens-1706778-supplementary/Figure S4 .pdf]
